# MR Angiography of the Head/Neck Vascular System in Mice on a Clinical MRI System

**DOI:** 10.1155/2019/5461809

**Published:** 2019-05-29

**Authors:** Carolin Reimann, Julia Brangsch, Lisa Christine Adams, Christa Thöne-Reineke, Bernd Hamm, Marcus Richard Makowski

**Affiliations:** ^1^Department of Radiology, Charite—Universitätsmedizin Berlin, Charitéplatz 1, 10117 Berlin, Germany; ^2^Department of Veterinary Medicine, Institute of Animal Welfare, Animal Behavior and Laboratory Animal Science, Freie Universität Berlin, Königsweg 67, Building 21, 14163 Berlin, Germany; ^3^King's College London, Division of Imaging Sciences and Biomedical Engineering, St Thomas' Hospital, Westminster Bridge Road, London SE1 7EH, UK

## Abstract

**Background:**

Magnetic resonance angiography (MRA) represents a clinical reference standard for the in vivo assessment of the vasculature. In this study, the potential of non-contrast-enhanced and contrast-enhanced angiography of the head/neck vasculature in mice on a clinical MR imaging system was tested.

**Methods:**

All in vivo magnetic resonance imaging was performed with a 3T clinical system (Siemens). Non-contrast-enhanced (time-of-flight, TOF) and contrast-enhanced angiography (gadofosveset-trisodium, GdT) were performed in C57BL/6J mouse strain. Lumen-to-muscle ratios (LMRs) and area measurements were assessed. Histology was performed as reference standard of all relevant vascular structures.

**Results:**

A close correlation between TOF (*R* ^2^ = 0.79; *p* < 0.05) and contrast-enhanced (GdT) angiography (*R* ^2^ = 0.92; *p* < 0.05) with histological area measurements was found. LMRs were comparable between both sequences. Regarding interobserver reproducibility, contrast-enhanced (GdT) angiography yielded a smaller 95% confidence interval and a closer interreader correlation compared to non-contrast-enhanced (TOF) measurements (−0.73–0.89; *R* ^2^ = 0.81 vs. −0.55–0.56; *R* ^2^ = 0.94).

**Conclusion:**

This study demonstrates that non-contrast-enhanced and contrast-enhanced angiographies of the head/neck vasculature of small animals can reliably performed on a clinical 3T MR scanner. Contrast-enhanced angiography enables the visualization of vascular structures with higher intravascular contrast and higher reproducibility.

## 1. Introduction

The most commonly used small animal models for scientific research are based on specific strains of mice [1]. After genetic modification, through surgical intervention or medical therapy, animal models allow for the investigation of specific pathological processes on a morphological or a molecular scale [2–4]. Most novel drug developments are initially tested in experimental animal models [5]. Especially in vascular diseases, noninvasive imaging modalities are more and more used to monitor changes in vivo, e.g. luminal stenosis in the case of atherosclerosis or luminal dilation in the case of aortic aneurysms [6, 7]. Traditionally, histological methods are applied to assess these differences. Such an approach is however associated with a relatively large number of animals required, as disease development cannot be studied longitudinally.

Regarding noninvasive imaging, magnetic resonance angiography (MRA) is an important clinical and preclinical imaging technique to assess changes in vascular dimensions noninvasively [8]. While X-ray angiography remains the reference standard for the visualization of the vascular system [9], due to its unique temporal and spatial resolutions, other imaging techniques, such as MR angiography, have significantly improved over the past years [10]. Different types of angiographic MR techniques exist. These include non-contrast-enhanced and contrast-enhanced techniques, both with their specific advantages and disadvantages [11–13]. In a clinical setting, the most established approaches include non-contrast-enhanced time-of-flight (TOF) technique and contrast-enhanced T1-weighted imaging techniques.

In experimental small animal studies, the use of noninvasive imaging modalities and especially MRI is not as widespread as it could be. Currently, most MRI studies in small animals are performed in dedicated preclinical MRI scanners, which usually work with higher field strengths (up to 16.4 Tesla) [14]. Most institutions do not have these dedicated systems available, as they require specific knowledge and a dedicated infrastructure with researchers with knowledge in pulse programming to maintain and run the systems. In comparison, clinical 3T MRI scanners are more available and provide an up-to-date sequence design, facilitating the translation of preclinical findings to clinical studies with human applications [15]. Due to significant developments in the hardware design including gradients strengths and receiver coils sensitivity, as well as in MR sequence design it became possible to scan at very high resolutions using clinical MRI systems.

The present study aims to assess the reliability and potential of MR angiography for the evaluation of the head/neck vasculature on a clinical 3T MRI system in the most widely used small animal mouse strain. We specifically tested the advantages and disadvantages of non-contrast-enhanced and contrast-enhanced angiography.

## 2. Methods

### 2.1. Setup of Animal Experiments

For this study, eight homozygous C57BL/6J mice aged 25 weeks (male) from the Charles Rivers Laboratories (Sulzfeld, Germany) were used. All procedures were approved by the guidelines and regulations of the Federation of Laboratory Animal Science Associations (FELASA) and the local Guidelines and Provisions for Implementation of the Animal Welfare Act. All animal procedures in this study were conducted by a veterinarian, and all possible steps were taken to avoid animal suffering at each stage of the experiment. The animals were fed with a standard lab diet and housed in a clean barrier. For the performed MRI imaging sessions, mice were anesthetized with an intraperitoneal administered combination of different drugs (500 *μ*g/kg Medetomidin, 50 *μ*g/kg Fentanyl, and 5 mg/kg Midazolam). At the end of the experimental procedures, the animals were sacrificed for histopathology. Mice were euthanized and exsanguinated in anterior perfusion with sodium chloride 0.9%. This was followed by a perfusion with the fixative MorFFFix® by Morphisto. The vessels were harvested for histological analysis. The aorta, brachiocephalic artery, and carotid artery were excised to allow anatomical matching.

### 2.2. Animal Handling and In Vivo Magnetic Resonance Imaging

Magnetic resonance data were collected using a clinical 3T Siemens mMR system (Siemens Healthcare Solutions, Erlangen, Germany). The anesthetized animals were placed in the MRI scanner in a prone position on a clinical single loop coil (47 mm, Siemens Healthcare Solutions, Erlangen, Germany). A gradient system with a gradient strength of 45 mT/m and a slew rate of 200 T/m/s was used. For sequence acquisition, a routine software package for vascular imaging was employed. The imaging protocol included the following sequences, which are also used in a clinical setting. At the beginning of the MR imaging protocol a low-resolution scout sequence (three-dimensional gradient echo sequence) was implemented to provide an anatomical overview of the localization of the vessels. In the next step, a transverse orientated non-contrast-enhanced two-dimensional time-of-flight angiography (2D TOF) was acquired for a precise visualization of the vessels. The time-of-flight MR angiography was performed with the following image parameters: slice thickness = 0.5 mm, in-plane spatial resolution = 0.035 × 0.035 mm^2^, imaging matrix = 576 × 576, field of view = 20 × 20 mm^2^, flip angle = 90°, echo time (TE) 3.8 ms, repetition time (TR) sequence = 57 ms, and scan time = 5 : 12 min. A maximum intensity projection (MIP) was automatically reconstructed based on the time-of-flight angiography. Following the acquisition of the non-contrast-enhanced time-of-flight angiography, 0.03 mmol/kg of clinically approved gadofosveset trisodium (GdT, Lantheus Medical Imaging, North Billerica, Massachusetts, USA) was administered via a catheter in the tail vein of the mouse. The delay between the administration of the contrast agent and the start of the acquisition was 10 minutes. Afterwards, a contrast-enhanced inversion recovery- (IR-) based MR angiography with the following imaging parameters was performed. The image parameters include slice thickness = 0.5 mm, in-plane spatial resolution = 0.13 × 0.13 mm^2^, imaging matrix = 416 × 416, field of view = 57 × 57 mm^2^, flip angle = 60°, echo time (TE) 7.7 ms, repetition time (TR) sequence = 37 ms, and scan time = 4 : 56 min. To facilitate small animal imaging research, we believe that a relatively short imaging protocols have to be established and tested. Therefore, we chose comparable scan times between the two sequences.

### 2.3. Histological Analysis of the Arterial Vessel System

Immediately after the MRI session, the animals were sacrificed for histological analysis. The histological analysis was performed for the region of aorta, the brachiocephalic artery, and carotid artery. Based on in vivo MR images, morphometric ex vivo data could thus be compared to the same regions. The surgically removed vessels were processed overnight. Subsequently, the vessels were embedded in paraffin and were cut into 5 *μ*m thick serial sections. After deparaffining and rehydration, the sections were stained with Miller's Elastica van Gieson stain (EvG) and hematoxylin and eosin (HE).

### 2.4. Magnetic Resonance Image Analysis

Measurements of the luminal signal were performed at the location of the aorta, brachiocephalic artery, and carotid artery. The analysis of all DICOM MR images was performed using Visage Imaging (version 7.1.7.1184, Visage Imaging, Inc.). For the evaluation of signal intensities, region of interests (ROIs) were drawn to delineate areas of signal enhancement on non-contrast-enhanced time-of-flight angiography and contrast-enhanced angiography following the administration of gadofosveset trisodium (GdT). Based on these regions of interest, lumen-to-muscle ratio (LMR) was calculated with following formula:

Lumen-to-muscle ratio of MR angiography = signal (vessel lumen)/signal (muscle).

Signal-to-noise ratio of MR angiography = signal (vessel lumen)/standard deviation signal.

This approach allows to compare between on non-contrast-enhanced time-of-flight angiography and contrast-enhanced angiography following the administration of gadofosveset trisodium (GdT).

### 2.5. Morphometry of the Head/Neck Arterial System

The complete arterial system including the aortic arch, ranging from the aortic bulbus to the descending aorta, subclavian arteries, brachiocephalic artery, and carotid arteries was visualized in all MR scans. The vessel bifurcations, e.g., the aorta to brachiocephalic artery and the brachiocephalic artery to subclavian artery, were used as anatomical landmarks for coregistration of the in vivo MR images and ex vivo images from histology. The morphometrical analysis was performed using elastin-stained sections (Miller's Elastica van Gieson stain) and ImageJ software (Version 1.51f, ImageJ).

### 2.6. Intra/Interobserver Agreements for Non-Contrast-Enhanced and Contrast-Enhanced MR Angiography

For the assessment of interobserver agreements images for non-contrast-enhanced and contrast-enhanced MR angiography, all acquired sequences were analyzed independently in a randomized order and blinded according to other imaging modalities by two independent readers. Area size was recorded for each measurement. For the assessment of intraobserver agreements images, all acquired sequences were analyzed in a randomized order and blinded according to other imaging modalities with a time difference of at least one month.

### 2.7. Statistical Analysis

A Student's *t*-test (two-tailed, unpaired) was used to compare continuous variables and to check the statistical significance between MRI and histological measurements. Linear regression was used to determine the relationship between measurements on MRI and histology. Interobserver agreements for in vivo measurements were assessed using Bland–Altman plots, which were generated for the raw volume data to indicate the spread of data and the limits of the agreement. The data are shown as mean ± standard deviation. A *p* value <0.05 was considered statistically significant.

## 3. Results

### 3.1. Comparison of Arterial Luminal Area Measurements of Non-Contrast-Enhanced and Contrast-Enhanced MR Angiography

**Figure 1:**
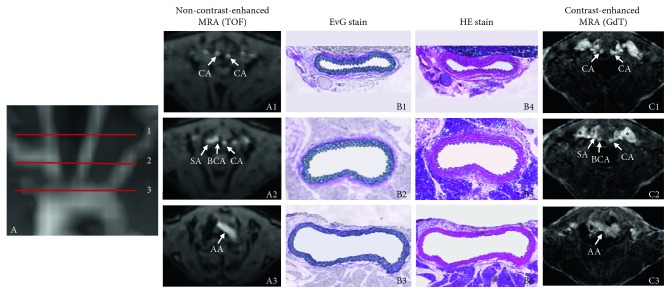
Visualization of the head/neck vasculature in a C57BL/6J mouse by MR angiography on a clinical 3T MR system. Images demonstrate the visualization of aorta (A3, B3, B6, C3), brachiocephalic artery (A2, B2, B5, C2), and carotid artery (A1, B1, B4, C1) in a C57BL/6J mouse by in vivo MR angiography on a clinical 3T MR system. Non-contrast-enhanced MR angiogram (TOF, A1-3) and contrast-enhanced MR angiogram (GdT, C1-3) in direct comparison to the associated histology of the arterial vessels (B). It must be highlighted that using an intravascular contrast agent, both arteries and adjacent veins are imaged of a high signal. The maximum intensity projection of the non-contrast-enhanced MR angiogram (A) indicates the location of transverse slices and vessels based on the red lines (1 = carotid artery, 2 = brachiocephalic artery, and 3 = aorta). Corresponding ex vivo histological sections (B1–B6), Elastica van Gieson (EvG) stain (B1, B2, B3), and hematoxylin eosin (HE) stain (B4, B5, B6) are demonstrated. CA: carotid artery, SA: subclavian artery, BCA: brachiocephalic artery, AA: aortic arch.

**Figure 2:**
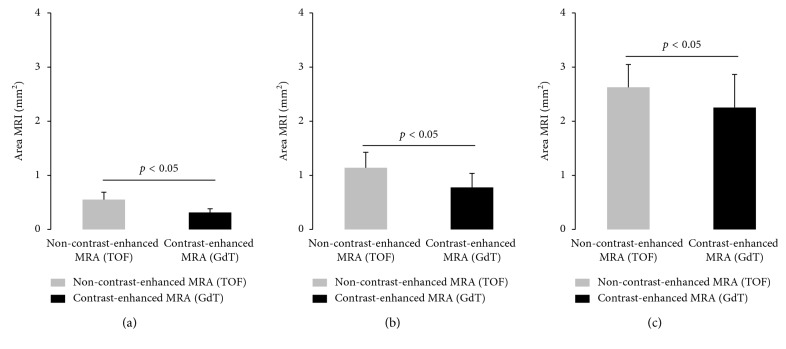
Area measurements of the aorta, brachiocephalic artery, and carotid artery on non-contrast-enhanced and contrast-enhanced MR angiographies. The diagram presents the luminal area measurements of the aorta, brachiocephalic artery, and carotid artery in mm^2^. Luminal area measurements based on the non-contrast-enhanced MR angiography (TOF) demonstrated significantly higher area measurements for the carotid artery (0.55 ± 0.14 mm^2^; *p* < 0.05), the brachiocephalic artery (1.14 ± 0.29 mm^2^; *p* ≤ 0.05), and the aorta (2.63 ± 0.42 mm^2^; *p* ≤ 0.05) compared to the contrast-enhanced MR angiography (GdT). Luminal area measurements for the contrast-enhanced MR angiography following the administration of gadofosveset trisodium were as follows: carotid artery (0.31 ± 0.07 mm^2^), brachiocephalic artery (0.78 ± 0.26 mm^2^), and aorta (2.25 ± 0.61 mm^2^). (a) MRA CA. (b) MRA BCA. (c) MRA aorta.

In non-contrast-enhanced MR angiography (TOF), luminal area measurement for the carotid artery (0.55 ± 0.14 mm^2^; *p* ≤ 0.05), the brachiocephalic artery (1.14 ± 0.29 mm^2^; *p* ≤ 0.05), and the aorta (2.63 ± 0.42 mm^2^; *p* < 0.05) were significantly larger compared to contrast-enhanced MR angiography (GdT, Figure 1). Luminal area measurements for contrast-enhanced MR angiography following the administration of gadofosveset trisodium were as follows: carotid artery (0.31 ± 0.07 mm^2^), brachiocephalic artery (0.78 ± 0.26 mm^2^), and aorta (2.25 ± 0.61 mm^2^). A summary of the results is presented in Figure 2.

### 3.2. Comparison of Lumen-to-Muscle Ratio Measurements of Non-Contrast-Enhanced and Contrast-Enhanced MR Angiography

For the assessment of the signal from the vascular lumen, all lumen-to-muscle ratio (LMR) measurements were based on the same regions of interest, which were used for the measurement of the vascular areas.

**Figure 3:**
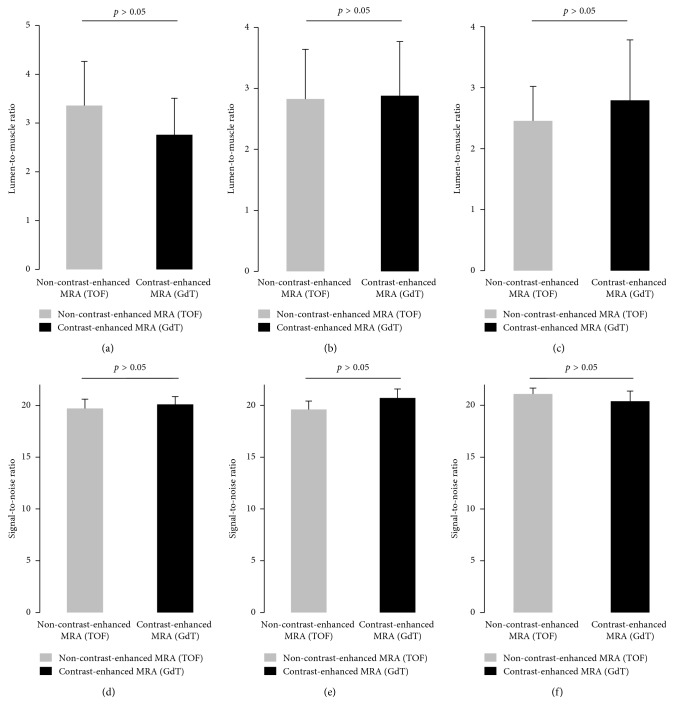
Lumen-to-muscle ratio and signal-to-noise measurements on of the aorta, brachiocephalic artery, and carotid artery on non-contrast-enhanced and contrast-enhanced MR angiographies. Lumen-to-muscle ratio (LMR) and signal-to-noise ratio (SNR) measurements were performed in the same animal at the same location in both non-contrast-enhanced magnetic resonance angiography (MRA) time-of-flight (TOF) and contrast-enhanced magnetic resonance angiography (MRA) gadofosveset-trisodium (GdT) images. For the aorta, lumen-to-muscle ratio measurements yield higher value for the non-contrast-enhanced MRA (TOF) compared to the contrast-enhanced MRA (GdT) (3.36 ± 0.91 vs. 2.76 ± 0.75). Difference did not reach statistical significance (*p* > 0.05). For the brachiocephalic artery, LMR values were comparable between the non-contrast-enhanced and contrast-enhanced MRA (2.83 ± 0.82 vs. 2.88 ± 0.89). For the carotid artery, the LMR of the non-contrast-enhanced MRA was lower compared to the contrast-enhanced MRA (2.45 ± 0.56 vs. 2.79–0.99). Difference in LMRs and SNRs between the two techniques was not significantly different (*p* > 0.05).

For the aorta, lumen-to-muscle ratio measurements yielded higher values for non-contrast-enhanced (TOF) compared to contrast-enhanced MRA (GdT) (3.36 ± 0.91 vs. 2.76 ± 0.75). However, this difference did not reach statistical significance (*p* > 0.05). For the brachiocephalic artery, LMR values were comparable between the non-contrast-enhanced and contrast-enhanced MRA (2.83 ± 0.82 vs. 2.88 ± 0.89). For the carotid artery, the LMR of the non-contrast-enhanced MRA was lower compared to the contrast-enhanced MRA (2.45 ± 0.56 vs. 2.79–0.99). Difference in digital area did not reach statistical significance (*p* > 0.05). A summary of the results is presented in Figure 3.

### 3.3. Comparison of Lumen Measurements of Non-Contrast-Enhanced and Contrast-Enhanced MR Angiography with Histology

**Figure 4:**
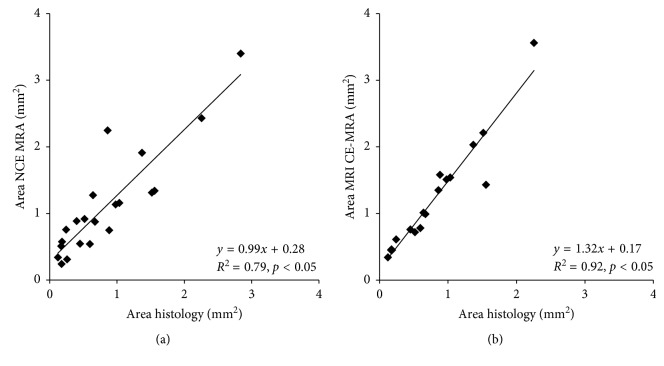
Correlation of non-contrast-enhanced and contrast-enhanced MR angiography with ex vivo histology. Both the non-contrast-enhanced (TOF) (A) and contrast-enhanced (GdT) (B) MR angiography, which were acquired on a clinical MRI system, enabled a reliable differentiation of the different artery types (carotid artery, brachiocephalic artery, and aorta). In vivo measurements of the non-contrast-enhanced and contrast-enhanced MR angiography were correlated with measurements on ex vivo histology (Elastica van Gieson stain, reference standard). Area measurements on in vivo contrast-enhanced MR angiograms (GdT) showed a close correlation with ex vivo measurements (*R* ^2^ = 0.92; *p* ≤ 0.05) (B), while in vivo measurements slightly and systemically overestimated the size of the vessel area (Figure 4), probably due to the well-known shrinkage of tissues due to the histological preparation. In non-contrast-enhanced MR angiography, area measurements showed a slightly lower correlation (*R* ^2^ = 0.79; *p* < 0.05) (A) with ex vivo histology compared to the contrast-enhanced MR angiography.

Both the non-contrast-enhanced (TOF) and contrast-enhanced (GdT) MR angiography, which were acquired on a clinical MRI system, enabled a reliable differentiation of the different artery types (carotid artery, brachiocephalic artery, and aorta). In in vivo measurements, the non-contrast-enhanced and contrast-enhanced MR angiography were correlated with measurements in ex vivo histology (Elastica van Giesson stain, reference standard). Area measurements in in vivo contrast-enhanced MR angiograms (GdT) showed a close correlation with ex vivo measurements (*R* ^2^ = 0.92; *p* < 0.05), while in vivo measurements slightly and systemically overestimated the size of the vessel area (Figure 4), probably due to the well-known shrinkage of tissues due to the histological preparation. In non-contrast-enhanced MR angiography, area measurements showed a slightly lower correlation (*R* ^2^ = 0.79; *p* < 0.05) with ex vivo histology compared to the contrast-enhanced MR angiography.

### 3.4. Interobserver and Intraobserver Agreements of Non-Contrast-Enhanced and Contrast-Enhanced MR Angiography

**Figure 5:**
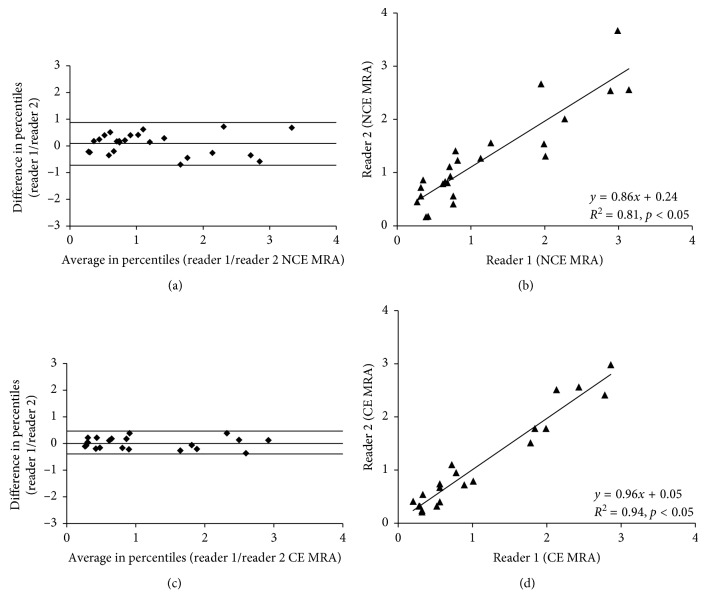
Interobserver agreements of non-contrast-enhanced and contrast-enhanced MR angiography. Interobserver correlation for area measurements in the non-contrast-enhanced MR angiography (TOF) showed a close correlation between both image readers (*R* ^2^ = 0.81; *p* < 0.05). The associated 95% confidence interval (CI) for the correlation range was −0.73 to 0.89. Interobserver correlation for area measurements for the contrast-enhanced MR angiography (GdT) showed a stronger correlation between both readers (*R* ^2^ = 0.94; *p* < 0.05). The associated 95% confidence interval (CI) for the correlation range was −0.42 to 0.43 (Figure 5).

Interobserver correlation for area measurements in the non-contrast-enhanced MR angiography (TOF) showed a close correlation between both image readers (*R* ^2^ = 0.81; *p* < 0.05). The associated 95% confidence interval (CI) for the correlation range was −0.73 to 0.89. Interobserver correlation for area measurements for the contrast-enhanced MR angiography (GdT) showed a stronger correlation between both readers (*R* ^2^ = 0.94; *p* < 0.05). The associated 95% confidence interval (CI) for the correlation range was −0.42 to 0.43 (Figure 5).

**Figure 6:**
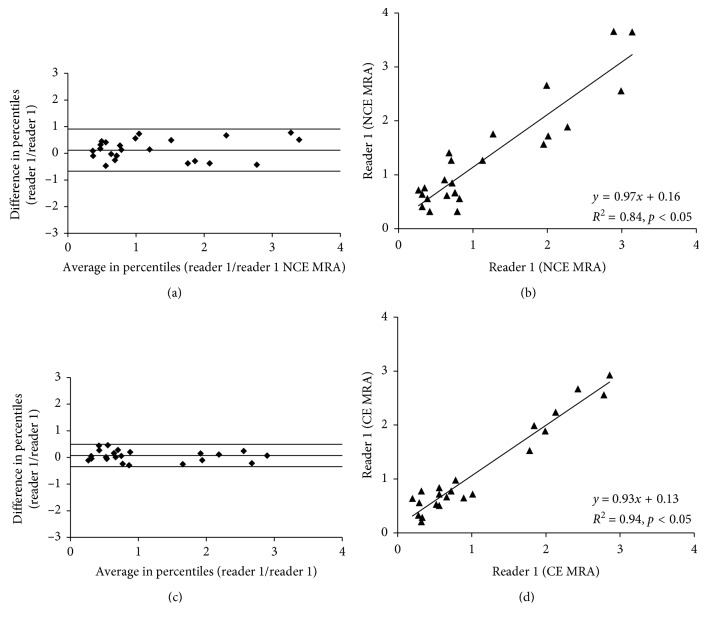
Intraobserver agreements of non-contrast-enhanced and contrast-enhanced MR angiography. The non-contrast-enhanced MRA showed a close correlation for the intraobserver agreement (*R* ^2^ = 0.84, *p* < 0.05). The associated confidence intervals ranged from −0.64 to 0.91. For the contrast-enhanced MRA, a stronger correlation was found (*R* ^2^ = 0.94). The associated 95% confidence intervals ranged from −0.37 to 0.48 (Figure 6).

Comparable measurements were derived for the intraobserver agreements. The non-contrast-enhanced MRA showed a close correlation for the intraobserver agreement (*R* ^2^ = 0.84, *p* < 0.05). The associated confidence intervals ranged from −0.64 to 0.91. For the contrast-enhanced MRA, a stronger correlation was found (*R* ^2^ = 0.94). The associated 95% confidence intervals ranged from −0.37 to 0.48 (Figure 6).

## 4. Discussion

This study demonstrates that MR angiography of the mouse head/neck vascular system can be reliably be performed on a clinical 3T MRI scanner. Both non-contrast-enhanced and contrast-enhanced techniques enable a reliable visualization of the head and neck arteries, including the aortic arch, brachiocephalic artery, and carotid artery in a short scan time. Contrast-enhanced MR angiography enables the visualization of the head and neck arterial system with a higher spatial resolution compared to non-contrast-enhanced techniques in the same imaging time. In vivo area measurements of both, the non-contrast-enhanced and contrast-enhanced MR angiography, showed a close correlation with ex vivo measurements on histology. Both techniques enabled imaging with a high interreader reproducibility, with contrast-enhanced MR angiography measurements showing a closer interreader agreement than non-contrast-enhanced imaging.

### 4.1. MR Angiography for Imaging of the Head/Neck Vascular System in an Experimental Setting

For the visualization of the head/neck vasculature, different MR angiographic techniques can be used [16, 17]. The most frequently used and clinically established MR sequences include non-contrast-enhanced and contrast-enhanced sequence techniques [17]. In an experimental setting, but also in a clinical setting, both techniques have their unique advantages and disadvantages.

The main advantage of non-contrast-enhanced techniques includes that the imaging contrast for the visualization of the vascular lumen can be generated without the need for contrast agents, which must be administered, e.g., via the tail vein. In this context, the most frequently used sequence design is based on a time-of-flight (TOF) technique. The TOF technique is based on the use of relatively short MR repetition times [18]. At each acquisition step, fresh or unsaturated nuclear spins flow into the imaging slice driven by the arterial blood stream. This results in the bright luminal signal in this MR angiographic technique. In the clinical setting, this imaging technique is routinely used for the visualization of cerebral arteries [18]. From a practical point of view, unenhanced imaging has the advantage of not requiring a venous access, which can be challenging to establish, especially if frequent longitudinal follow-up investigations are part of the experimental setup. An additional advantage is that there is no risk to miss the first pass of the contrast agent. Especially in small animals, the acquisition of first pass angiography is highly challenging, as the high heart rate of mice requires imaging within extremely high temporal resolution. A further advantage of non-contrast-enhanced techniques is that there is no risk of interference with, e.g., the biokinetics of a potentially tested molecular probe.

The main advantage of contrast-enhanced angiographic MR techniques is that due to the high intravascular contrast, a high spatial resolution of the vascular lumen can be achieved in a short imaging time. The luminal imaging contrast in this type of sequence depends on the T1 shortening effect of the contrast agent. In a clinical setting, the most frequently used contrast agents are extravascular contrast agents. This type of contrast agent has a relatively short blood half-life and quickly diffuses into the interstitial space. Therefore, MR angiographies are performed during the first pass of the contrast agent directly following the intravenous administration. For imaging of small animals in a clinical MR system, the performance of first pass MR angiographies can however be challenging, as the first pass of the agent can be missed. Another type of contrast agent that is available for MR angiographies are intravascular contrast agents, which show a relatively long blood half-life and therefore enables imaging for a prolonged period. In the current study, the intravascular contrast agent gadofosveset trisodium was used. This probe binds to serum albumin, resulting in an increase of its R1 relaxivity and thereby a strong intravascular contrast. For imaging of small animals, the long intravascular half-life is especially advantageous, as imaging can be performed for a prolonged time frame and independent of the first pass. Disadvantages of the use of intravascular agents in combination include the low contrast between arteries and adjacent veins. In the current study, the potential of non-contrast-enhanced and contrast-enhanced angiography for the visualization of the head/neck vasculature in small animals was tested. A direct comparison of both imaging techniques in the same animal was performed. The C57BL/6J mouse strain, which is the most widely used mouse type in the research community, was used. Using the non-contrast-enhanced time-of-flight (TOF) angiographic technique, a lower spatial resolution, compared to surrounding tissues, could be achieved. However, the large head/neck vascular structures which include the aorta, brachiocephalic artery, subclavian artery, and carotid arteries could be reliably visualized. This could be important for the study design of preclinical studies, e.g., using molecular probes. The time-of-flight technique seems to be especially well suited for these types of studies, as based on this sequence a reliable colocalization of the signal from, e.g., probes in the vascular wall can be achieved. In vivo vascular size measurements correlated well with ex vivo measurements on histology. Additionally, a high interobserver reproducibility could be accomplished for these measurements.

Using contrast-enhanced angiographic techniques, a relatively high spatial resolution with a high intravascular contrast could be achieved, compared to the surrounding tissue. Even small vascular structures, including the carotid arteries, could be visualized with a high interobserver reproducibility. A strong correlation between in vivo and ex vivo measurements could be measured. This indicates that this type of sequence might be more useful for, e.g., the quantification of the vascular diameter compared to non-contrast-enhanced time-of-flight techniques. The main disadvantages of this approach include that the administration of a contrast agent is required.

### 4.2. Preclinical versus Clinical Magnetic Resonance Systems in an Experimental Setting

Currently, most MRI studies in small animals were performed using dedicated preclinical MRI scanners. These systems usually have high or even ultrahigh field strengths (up to 16.4 Tesla). However, most institutions do not have these dedicated systems available, as they usually require dedicated researchers with knowledge in pulse programming to maintain and run the systems. Due to significant developments in the hardware (e.g., gradients and receiver coils) and in the MR sequence design, it is also possible to scan at very high resolutions using routine clinical MRI systems.

A strength of this study was that all imaging was performed at a clinical field strength (3 Tesla). Most experimental small animal studies were performed at dedicated high field or ultrahigh field preclinical systems (e.g., 9.4 Tesla). Even though a high signal-to-noise ratio (SNR) and carrier-to-noise-ratio (CNR) can be achieved using these systems, the magnetic properties of gadolinium-based T1 shortening probes are significantly altered by such a high field strength. Therefore, findings at these high field strengths cannot be easily translated into a clinical setting. Additionally, the contrast agent gadofosveset trisodium in this study was developed to exhibit its highest relaxivity at clinical field strength [19]. A further advantage of clinical MR systems is that highly developed clinically validated MR imaging sequences are available which can be scaled down for the use in small animals.

### 4.3. Limitations

Clinical MRI scanners with a clinically used field strength (1.5–3.0 T) yield a lower signal-to-noise ratio (SNR) compared scanner with a high field strength (4.7–16.4 T). However, in the present study, we did not perform a comparison between ultrahigh field dedicated preclinical MR imaging systems and clinical MR systems. The production of Gadofosveset has been currently discontinued. The contrast agent is still available for research use; however, for clinical use, no comparable alternative for intravascular extracellular contrast agent with such a long blood half-life time is available. A further limitation of this study is the lack of experiments in animal models of pathologies.

## 5. Conclusion

This study demonstrates that non-contrast-enhanced and contrast-enhanced angiographies of the head/neck vasculature of small animals can be reliably performed on a clinical 3T MR scanner. Contrast-enhanced angiography enables the visualization of vascular structures with a comparably higher intravascular contrast and higher reproducibility.

## Data Availability

The datasets generated and analysed during the current study are not publicly available but are available from the corresponding author on reasonable request.

## Supplementary Materials

Supplementary Materials Supplementary Figure 1: visualization of an abdominal aortic aneurysm (AAA) and in ApoE–/– knockout mouse by MR angiography on a clinical 3T MR system. Images demonstrate the visualization of AAA by in vivo MR angiography on a clinical 3T MR system. Non-contrast- enhanced MR angiogram (TOF, a) and contrast-enhanced MR angiogram (GdT, b) in direct comparison to the associated histology of the arterial vessels (c). A strong correlation between in vivo and ex vivo measurements was measured (4a).

## References

[1] Perlman R. L. (2016). Mouse models of human disease: an evolutionary perspective. *Evolution, Medicine, and Public Health*.

[2] Kersten K., de Visser K. E., van Miltenburg M. H., Jonkers J. (2017). Genetically engineered mouse models in oncology research and cancer medicine. *EMBO Molecular Medicine*.

[3] Breckenridge R. (2010). Heart failure and mouse models. *Disease Models & Mechanisms*.

[4] Tarnavski O. (2009). Mouse surgical models in cardiovascular research. *Methods in Molecular Biology*.

[5] Vandamme T. (2014). Use of rodents as models of human diseases. *Journal of Pharmacy and Bioallied Sciences*.

[6] Sannino A., Brevetti L., Giugliano G. (2014). Non-invasive vulnerable plaque imaging: how do we know that treatment works?. *European Heart Journal—Cardiovascular Imaging*.

[7] Hong H., Yang Y., Liu B., Cai W. (2010). Imaging of abdominal aortic aneurysm: the present and the future. *Current Vascular Pharmacology*.

[8] Oostendorp M., Post M. J., Backes W. H. (2009). Vessel growth and function: depiction with contrast-enhanced MR imaging. *Radiology*.

[9] Danias P. G., Roussakis A., Ioannidis J. P. A. (2004). Diagnostic performance of coronary magnetic resonance angiography as compared against conventional x-ray angiography. *Journal of the American College of Cardiology*.

[10] Upputuri P. K., Sivasubramanian K., Mark C. S., Pramanik M. (2015). Recent developments in vascular imaging techniques in tissue engineering and regenerative medicine. *BioMed Research International*.

[11] Wheaton A. J., Miyazaki M. (2012). Non-contrast enhanced MR angiography: physical principles. *Journal of Magnetic Resonance Imaging*.

[12] Miyazaki M., Lee V. S. (2008). Nonenhanced MR angiography. *Radiology*.

[13] Runge V. M., Kirsch J. E., Lee C. (1993). Contrast-enhanced MR angiography. *Journal of Magnetic Resonance Imaging*.

[14] Marzola P., Osculati F., Sbarbati A. (2003). High field MRI in preclinical research. *European Journal of Radiology*.

[15] Farrar C. T., Dai G., Novikov M. (2008). Impact of field strength and iron oxide nanoparticle concentration on the linearity and diagnostic accuracy of off-resonance imaging. *NMR in Biomedicine*.

[16] Kobayashi H., Sato N., Hiraga A. (2001). 3D-micro-MR angiography of mice using macromolecular MR contrast agents with polyamidoamine dendrimer core with reference to their pharmacokinetic properties. *Magnetic Resonance in Medicine*.

[17] Evangelista A. (2014). Imaging aortic aneurysmal disease. *Heart*.

[18] MacDonald M. E., Frayne R. (2015). Cerebrovascular MRI: a review of state-of-the-art approaches, methods and techniques. *NMR in Biomedicine*.

[19] Overoye-Chan K., Koerner S., Looby R. J. (2008). EP-2104R: a fibrin-specific gadolinium-based MRI contrast agent for detection of thrombus. *Journal of the American Chemical Society*.

