# Supplementary material for: MR Angiography of the Head/Neck Vascular System in Mice on a Clinical MRI System
Source: Contrast Media Mol Imaging. 2019 May 29;2019:5461809. doi: 10.1155/2019/5461809 (PMC6560327; doi:10.1155/2019/5461809)
Supplement: Supplementary Materials — Supplementary Figure 1: visualization of an abdominal aortic aneurysm (AAA) and in ApoE–/– knockout mouse by MR angiography on a clinical 3T MR system. Images demonstrate the visualization of AAA by in vivo MR angiography on a clinical 3T MR system. Non-contrast- enhanced MR angiogram (TOF, a) and contrast-enhanced MR angiogram (GdT, b) in direct comparison to the associated histology of the arterial vessels (c). A strong correlation between in vivo and ex vivo measurements was measured (4a). [file 5461809.f1.pdf]

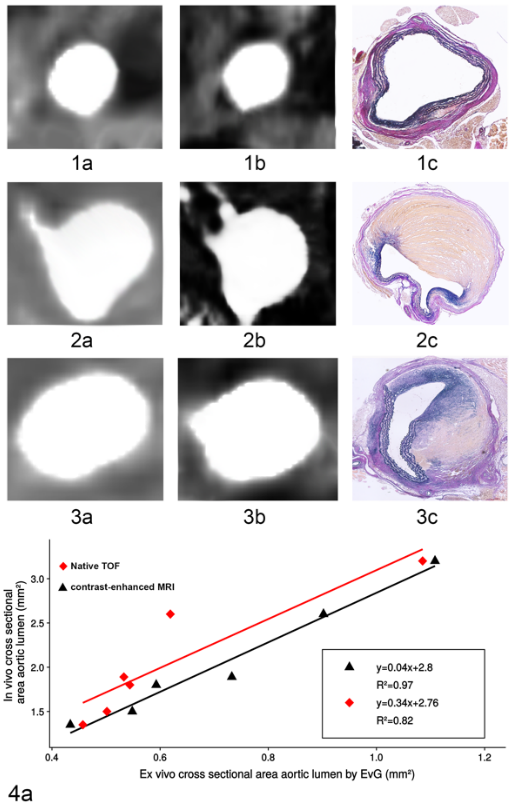

Supplementary Figure 1: Visualization of an abdominal aortic aneurysm (AAA) and in ApoE-/- knockout mouse by MR angiography on a clinical 3T MR system. Images demonstrate the visualization of AAA by in vivo MR angiography on a clinical 3T MR system. Non-contrast-enhanced MR angiogram (TOF, a) and contrast-enhanced MR angiogram (GdT, b) in direct comparison to the associated histology of the arterial vessels (c). A strong correlation between in vivo and ex vivo measurements was measured (4a).
